# 25-Hydroxyvitamin D, IL-31, and IL-33 in Children with Allergic Disease of the Airways

**DOI:** 10.1155/2014/520241

**Published:** 2014-06-26

**Authors:** Anna Bonanno, Sebastiano Gangemi, Stefania La Grutta, Velia Malizia, Loredana Riccobono, Paolo Colombo, Fabio Cibella, Mirella Profita

**Affiliations:** ^1^Institute of Biomedicine and Molecular Immunology “A. Monroy” (IBIM), National Research Council of Italy (CNR), Palermo, Italy; ^2^Department of Clinical and Experimental Medicine, School and Division of Allergy and Clinical Immunology, University of Messina, Messina, Italy

## Abstract

Low vitamin D is involved in allergic asthma and rhinitis. IL-31 and IL-33 correlate with Th2-associated cytokines in allergic disease. We investigated whether low vitamin D is linked with circulating IL-31 and IL-33 in children with allergic disease of the airways. 25-Hydroxyvitamin D [25(OH) Vit D], IL-31, and IL-33 plasma levels were measured in 28 controls (HC), 11 allergic rhinitis (AR) patients, and 35 allergic asthma with rhinitis (AAR) patients. We found significant lower levels of 25(OH) Vit D in AR and in AAR than in HC. IL-31 and IL-33 plasma levels significantly increased in AAR than HC. IL-31 and IL-33 positively correlated in AR and AAR. 25(OH) Vit D deficient AAR had higher levels of blood eosinophils, exacerbations, disease duration, and total IgE than patients with insufficient or sufficient 25(OH) Vit D. In AAR 25(OH) Vit D levels inversely correlated with total allergen sIgE score and total atopy index. IL-31 and IL-33 did not correlate with 25(OH) Vit D in AR and AAR. In conclusion, low levels of 25(OH) Vit D might represent a risk factor for the development of concomitant asthma and rhinitis in children with allergic disease of the airways independently of IL-31/IL-33 Th2 activity.

## 1. Introduction

The hormonal form of vitamin D affects both adaptive and innate immune functions involved in the development of allergies [1]. Genetic factors of the vitamin D metabolism are involved in the development of allergic conditions showing a direct association between vitamin D receptor polymorphisms and atopic asthma [2]. Furthermore, studies on an animal model provided evidence for a link between early vitamin D supplementation and the later allergy where several vitamin D regulated genes seem to be involved [3]. Recently, low serum vitamin D levels were observed in association with airway obstruction and corticosteroid requirement in asthmatic patients influencing the asthma severity, atopy, or both [4–6].

Vitamin D in the form of 1,25-dihydroxyvitamin D is a potent immune system modulator and at the molecular level has been shown to be involved in the suppression of dendritic cell maturation and consecutive Th1 cell development [7–9]. In fact, vitamin D may suppress the production of IL-12, thereby reducing the production of T helper type 1 (Th1) cells and potentially leading to increased proliferation of allergy-associated T helper type 2 (Th2) cells [7]. Additionally, studies in mice have shown that treatment with 1,25-dihydroxyvitamin D results in the reduced secretion of Th1-type cytokines IL-2 and IFN-*γ* and an increase in Th2-type IL-4 [10].

Interleukin 33 (IL-33) potently drives the production of proinflammatory Th2-associated cytokines, including IL-4, IL-5, and IL-13, by in vitro polarized Th2 cells [11], mast cells [12], basophils and eosinophils [13, 14], and dendritic cells [15]. Furthermore, hematopoietic cells produce other inflammatory cytokines and chemokines, including IL-6 and IL-8, via IL-33 stimulation [14]. These activities suggest potential roles for IL-33 in Th2-associated immune responses, and thus IL-33 is thought to be closely associated with allergic inflammatory diseases, including asthma. Indeed, increased IL-33 levels were observed in the bronchoalveolar lavage fluid from subjects with moderate asthma compared with mild asthmatics and controls without asthma [16]. A single-nucleotide polymorphism in IL-33 that showed a suggestive association with the circulating eosinophil count was also significantly associated with atopic asthma [17].

Interleukin 31 (IL-31) is a T helper type 2 effector cytokine that plays an important role in the pathogenesis of atopic and allergic diseases. IL-31, mainly produced by activated Th2 cells, interacts with a heterodimeric receptor consisting of IL-31 receptor A (IL-31RA) and the oncostatin M receptor (OSMR) constitutively expressed on epithelial cells and keratinocytes. Plasma IL-31 concentration was found to be significantly elevated in patients with atopic dermatitis (AD) compared to healthy individuals [18] and positively correlated with disease severity [19]. Mainly, a crucial immunopathological role of IL-31 and IL-33 was observed in AD suggesting their inflammatory effect in atopic disease [20].

The present study aims to investigate the plasma levels of 25(OH) Vit D, IL-31, and IL-33 in children with allergic asthma and rhinitis. Furthermore, since lower levels of 25(OH) Vit D and high levels of IL-31 and IL-33 are associated with Th2 immunity of allergic disease, we hypothesized a potential interaction between these markers and clinical and functional parameters in asthma and rhinitis.

## 2. Methods

### 2.1. Subjects

Pediatric subjects (age between 8 and 13 years) were recruited among outpatients attending the Pulmonology/Allergy Clinic of the National Research Council of Italy in Palermo. Asthma diagnosis and assessment of severity were performed according to Global Initiative for Asthma (GINA) guidelines [21]. AR diagnosis was performed at the study entry according to Allergic Rhinitis and its Impact on Asthma (ARIA) guidelines [22].

The patients were divided in two groups: 35 children had concomitant intermittent to persistent allergic rhinitis and intermittent to moderate allergic asthma (AAR), and 11 patients had intermittent to persistent allergic rhinitis (AR). The control group, having negative assessment of the atopic status and no asthma or rhinitis symptoms, was composed of 28 healthy children (HC). Children stopped the treatment for asthma (ICS) or rhinitis (antihistamines or topical steroid) 1 month before entering in the study.

No patients had nasal polyposis or bronchial or respiratory tract infections or had a severe exacerbation of asthma resulting in hospitalization during the last month. Within 2 days from blood samples, all subjects performed pulmonary function tests as recommended by the GINA guidelines.

The study was approved by the Ethics Committee of the University Hospitals of Palermo and complied with the Helsinki Declaration (N°7/2013). Written informed consent was obtained from the parents of the patients enrolled in the study.

### 2.2. Assessment of the Atopic Status

Skin prick tests were performed according to EAACI recommendations [23] with a standard panel including* Dermatophagoides* mix, grass mix,* Parietaria judaica*, olive, dog and cat dander,* Alternaria*, and* Blattella germanica*, plus a positive (histamine 1%) and a negative (saline) control (Stallergenes Italia S.r.l., Milan, Italy). The reading was performed after 15 min: reactions were considered positive if the mean wheal diameter (computed as the maximum diameter plus its orthogonal divided by two) was 3 mm or greater, after having subtracted the wheal diameter of the reaction to the negative control. Total and allergen-specific serum IgE levels were determined by CAP System (Pharmacia-Upjohn, Uppsala, Sweden). The total atopy index (degree of allergic sensitization) was also assessed, which is defined as the total number of allergens tested to which each subject had a positive response [24]. The total allergen sIgE score (sum of classes of allergen specific IgE test) was considered as a quantitative score able to reflect the levels of allergen exposure and the degree of multiple sensitizations in the patients [25]. Total eosinophil count was performed using standard procedures.

### 2.3. Measurement of Plasma 25(OH) Vit D by ELISA

Plasma 25(OH) Vit D was assayed using an enzyme linked immunosorbent assay (ELISA) kit (Immunodiagnostic System Ltd., Boldon Business Park, Boldon, Tyne and Wear, UK) according to the manufacturer instructions. The absorbance was read at 450 nm (reference 620 nm) by a Wallac 1420 Victor2 multilabel counter (Perkin-Elmer Life Sciences, Turku, Finland). This method had 100% and 75% specificity for 25(OH)D3 and 25(OH)D2, respectively, and assessed the overall vitamin D status but did not distinguish between these two forms [26]. The limit of detection was 2 ng/mL. The intra- and interassay coefficients of variation were 5.3% and 4.6%, respectively. Samples were run in duplicate with quality control samples to ensure day-to-day validity of results.

### 2.4. Measurements of Plasma IL-31 and IL-33 by ELISA

Plasma IL-31 and IL-33 protein levels were measured using the commercially available DuoSet ELISA Development System kits (R & D Systems; Minneapolis, MN, USA) specific for human IL-31 and human IL-33. All analyses were performed according to the manufacturer's protocol. Absorbance was measured at 450 nm (correction wavelength set at 540 nm) by a Wallac 1420 Victor2 multilabel counter (Perkin-Elmer Life Sciences, Turku, Finland). The detection limit was 15.6 pg/mL for IL-31 and 4.0 pg/mL for IL-33.

### 2.5. Statistical Analysis

Statistical analysis was performed using Kruskal-Wallis test and the differences between groups were evaluated by nonparametric Mann-Whitney *U* test. Correlation analyses were performed with Spearman's rank test. The chi-square (*χ* ^2^) analysis was performed to evaluate differences in frequency distributions of variables. Data were expressed as means ±SD or median values with interquartile ranges. A *P* value < 0.05 was considered statistically significant.

## 3. Results

### 3.1. Demographic Characteristics of Patients

We reported the demographic, clinical, and atopic characteristics of all patients included in the study in Table 1.

### 3.2. 25(OH) Vit D, IL-31, and IL-33 in Patients with AR and AAR

**Figure 1:**
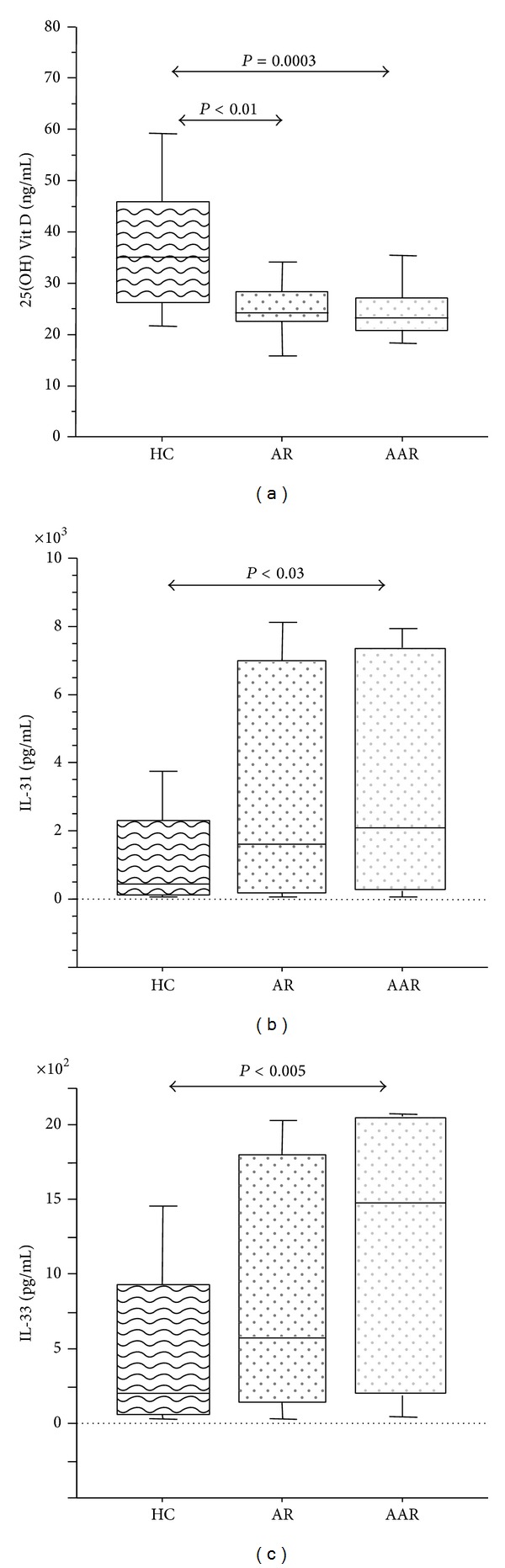
Plasma levels of 25(OH) Vit D (ng/mL), IL-31 (pg/mL), and IL-33 (pg/mL) in HC (*n* = 28) compared to patients with AR (*n* = 11) and patients with AAR (*n* = 35). 25(OH) Vit D concentrations (a), IL-31 concentrations (b), and IL-33 concentrations (c) were analyzed by specific ELISA. Bars in the box plots indicate (from the bottom to the top) 10th, 25th, 50th (median), 75th, and 90th percentiles. Statistical analysis was performed by Kruskal-Wallis test and Mann-Whitney *U* test. Significance was accepted at *P* < 0.05.

We found significant lower levels of 25(OH) Vit D in AR (median; range: 24.0 ng/mL, 22.5–28.3; *P* < 0.01) and in AAR (median; range: 23.5 ng/mL, 21.0–27.1; *P* = 0.0003) than in HC (median; range: 35.0 ng/mL, 26.3–46.0) (Figure 1(a)). IL-31 and IL-33 levels increased in AAR and AR than in HC (Figures 1(b) and 1(c)); the difference was statistically significant only between AAR and HC (IL-31: *P* < 0.03; IL-33: *P* < 0.005) although higher levels of IL-31 and IL-33 were observed in AR than in HC (Figures 1(b) and 1(c)).

In accordance with 25(OH) Vit D levels, we categorized subjects of each group enclosed in the study as deficient (def: <20 ng/mL), insufficient (insuff: ≥20 and <30 ng/mL), and sufficient (suff: ≥30 ng/mL) as previously described [27]. Frequency distribution of 25(OH) Vit D status classes (deficiency, insufficiency, and sufficiency) in HC, AR, and AAR groups is shown in Table 2. Differences in the distribution are significant (*P* < 0.009(*χ* ^2^)).

### 3.3. Correlation between 25(OH) Vit D, IL-31, and IL-33 in Patients with AR and AAR

**Figure 2:**
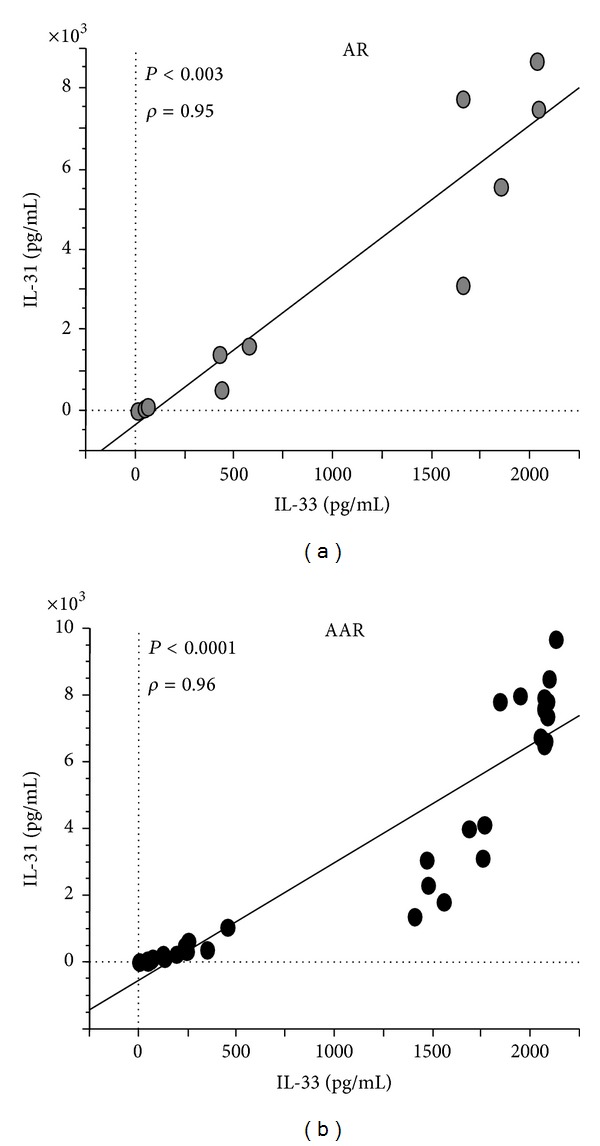
Correlation between plasma IL-31 and IL-33 concentrations in (a) AR patients (*n* = 11) and (b) AAR patients (*n* = 35). Statistical analysis was performed by Spearman rank test. Significance was accepted at *P* < 0.05.

In AR and AAR patients, no correlation was found between the 25(OH) Vit D levels and IL-31 or IL-33 levels (data not shown). However, IL-31 and IL-33 plasma levels showed a positive correlation in AR (*P* < 0.003) (Figure 2(a)) and in AAR patients (*P* < 0.0001) (Figure 2(b)).

### 3.4. 25(OH) Vit D Level, IL-31, IL-33, and Atopic Status in Patients with Allergic Diseases

**Figure 3:**
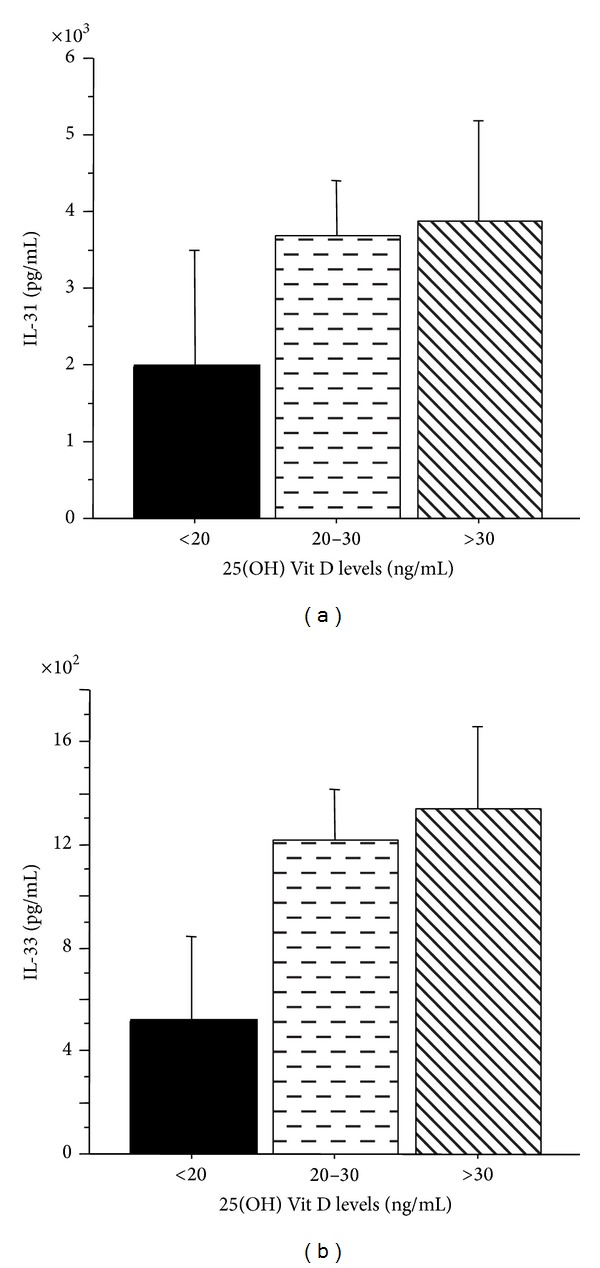
IL-31 and IL-33 plasma levels and 25(OH) Vit D status in patients with AAR (*n* = 35). (a) IL-31 concentrations and (b) IL-33 concentrations were measured by specific ELISA in AAR patients with deficient (*n* = 6), insufficient (*n* = 22), or sufficient (*n* = 7) 25(OH) Vit D levels. Bars represent mean ±SD. Statistical analysis was performed by Kruskal-Wallis test. Significance was accepted at *P* < 0.05.

**Figure 4:**
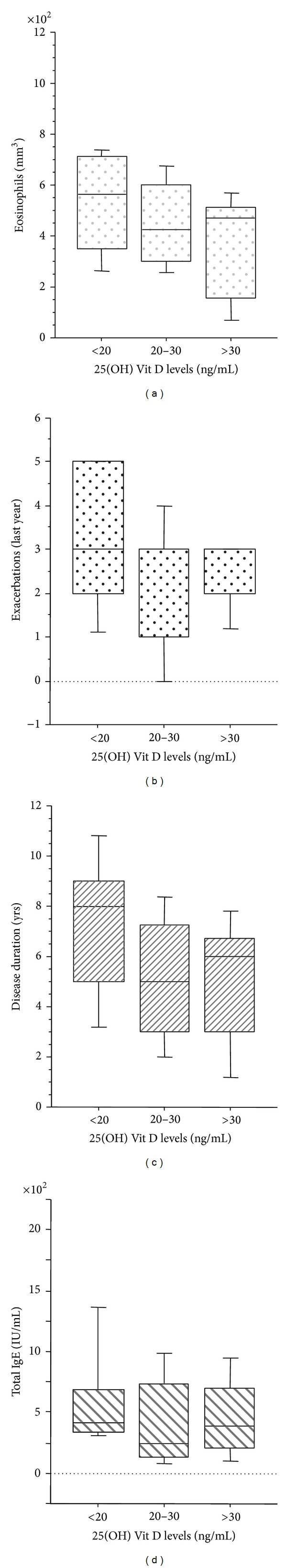
Asthma control parameters and 25(OH) Vit D status in patients with AAR (*n* = 35). (a) Blood eosinophils (number/mm^3^), (b) exacerbations (number/last year), (c) disease duration (yrs), and (d) total IgE (IU/mL) in AAR patients with deficient (*n* = 6), insufficient (*n* = 22), or sufficient (*n* = 7) 25(OH) Vit D levels. Bars in the box plots indicate (from the bottom to the top) 10th, 25th, 50th (median), 75th, and 90th percentiles. Statistical analysis was performed by Kruskal-Wallis test. Significance was accepted at *P* < 0.05.

**Figure 5:**
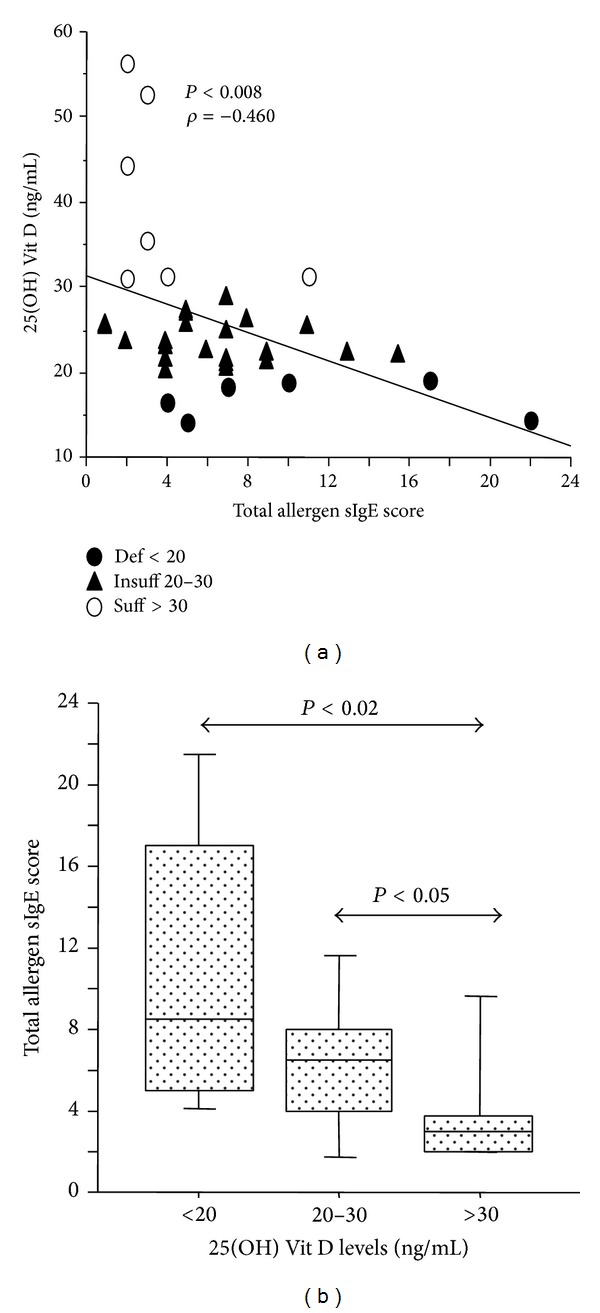
Relationship between 25(OH) Vit D plasma levels and total allergen sIgE score in patients with AAR (*n* = 35). (a) 25(OH) Vit D levels and total allergen sIgE score inversely correlated in AAR patients. Statistical analysis was performed by Spearman rank test. (b) Total allergen sIgE score in AAR patients with deficient (*n* = 6), insufficient (*n* = 22), or sufficient (*n* = 7) 25(OH) Vit D levels. Bars in the box plots indicate (from the bottom to the top) 10th, 25th, 50th (median), 75th, and 90th percentiles. Statistical analysis was performed by Kruskal-Wallis test and Mann-Whitney *U* test. Significance was accepted at *P* < 0.05.

**Figure 6:**
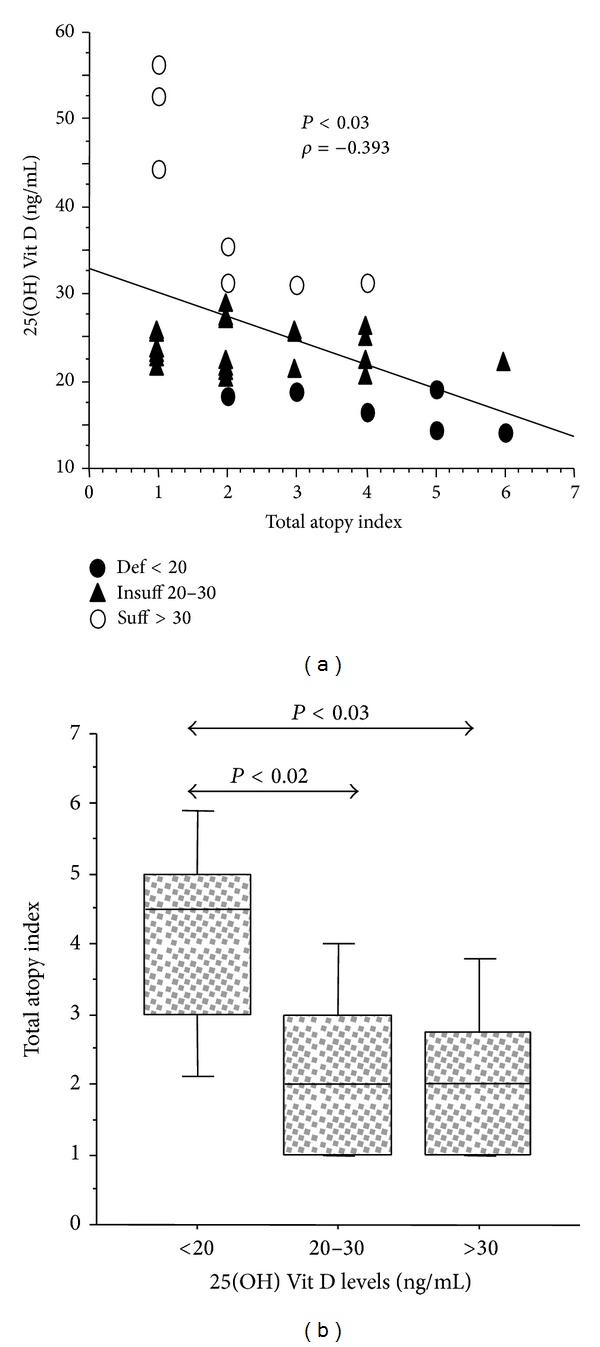
Relationship between 25(OH) Vit D plasma levels and total atopy index in patients with AAR (*n* = 35). (a) 25(OH) Vit D levels and total atopy index inversely correlated in AAR patients. Statistical analysis was performed by Spearman rank test; (b) total atopy index in AAR patients with deficient (*n* = 6), insufficient (*n* = 22), or sufficient (*n* = 7) 25(OH) Vit D levels. Bars in the box plots indicate (from the bottom to the top) 10th, 25th, 50th (median), 75th, and 90th percentiles. Statistical analysis was performed by Kruskal-Wallis test and Mann-Whitney *U* test. Significance was accepted at *P* < 0.05.

We analyzed plasma levels of IL-31 and IL-33 in AR and AAR patients grouped by 25(OH) Vit D status. IL-31 and IL-33 were not significantly different in AR and AAR patients showing deficient, insufficient, or sufficient 25(OH) Vit D levels. However, IL-31 and IL-33 levels were higher in AAR patients with insufficient or sufficient 25(OH) Vit D (Figure 3). Additionally, we analyzed clinical and atopic parameters in AR and AAR patients grouped by 25(OH) Vit D status. We observed in AAR, but not in AR group, that patients with deficient 25(OH) Vit D had higher levels of blood eosinophils (number/mm^3^), exacerbations (number/last year), disease duration (yrs), and total IgE (IU/mL) than patients with insufficient or sufficient 25(OH) Vit D without significant differences among the groups (Figure 4). Again, only in AAR group, 25(OH) Vit D levels inversely correlated with total allergen sIgE score (sum of classes of allergen specific IgE test) (*P* < 0.008) (Figure 5(a)) and patients with deficient or insufficient 25(OH) Vit D showed significant higher levels of total allergen sIgE score than those with sufficient 25(OH) Vit D (Figure 5(b)). Finally, in AAR group, 25(OH) Vit D levels inversely correlated with total atopy index (number of positive allergens) (*P* < 0.03) (Figure 6(a)) and patients with deficient 25(OH) Vit D showed significant higher levels of total atopy index than patients with insufficient or sufficient 25(OH) Vit D (Figure 6(b)). This finding clearly suggests that low levels of 25(OH) Vit D are associated with the degree of multiple allergen sensitizations only in patients with AAR.

**Figure 7:**
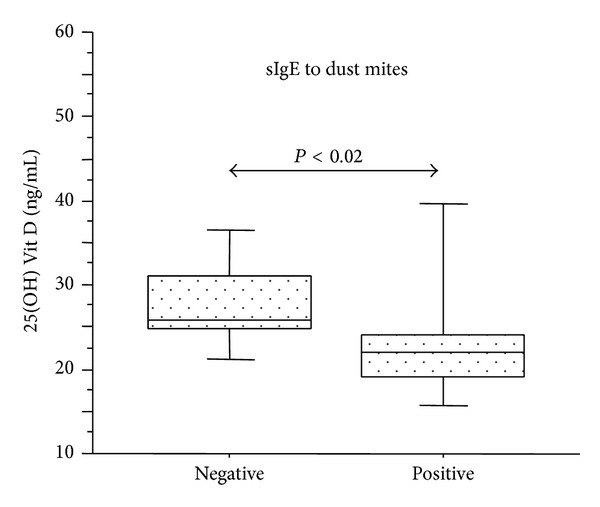
25(OH) Vit D levels and sIgE to dust mites in patients with AAR (*n* = 35). 25(OH) Vit D concentrations were measured by ELISA in AAR patients with negative sIgE to dust mites (*n* = 14) and in AAR patients with positive sIgE to dust mites (*n* = 21). Bars in the box plots indicate (from the bottom to the top) 10th, 25th, 50th (median), 75th, and 90th percentiles. Statistical analysis was performed by Mann-Whitney *U* test. Significance was accepted at *P* < 0.05.

AAR pediatric patients, showing positive sIgE to dust mites, had significant lower 25(OH) Vit D levels than children negative to dust mites sIgE (*P* < 0.02). The median concentrations of 25(OH) Vit D levels were 22.1 ng/mL (range: 19.1–24.1) in dust mite positive AAR children and 25.94 ng/mL (range: 24.9–31.1) in dust mite negative AAR children (Figure 7). The same differences were not observed in AR patients. No associations between positive sIgE levels to cat, dog,* Alternaria* species, tree mix, grass mix, and weed mix and 25(OH) Vit D levels were found (data not shown) in AAR and AR children.

## 4. Discussion

The present study investigated for the first time the relationship between IL-31, IL-33, and 25(OH) Vit D in pediatric allergic disease of the airways. We observed that although all these markers appear to be involved in allergic rhinitis and in concomitant allergic asthma and rhinitis, no correlations were observed between 25(OH) Vit D and IL-31 or IL-33. These results suggest that lower levels of 25(OH) Vit D affect allergic disease of the airways independently of the pathological role of IL-31 and IL-33. Finally, we observed that children with concomitant asthma and rhinitis, showing deficient 25(OH) Vit D, have higher levels of blood eosinophils, exacerbations in last year, disease duration, total allergen sIgE score, and total atopy index. These findings might underline that low levels of 25(OH) Vit D represent a major favorable outcome for children with allergic rhinitis to develop concomitant allergic asthma.

The knowledge on direct mechanistic links between vitamin D and lung diseases are limited. However, a number of epidemiological and experimental available data highlight the relevance of this connection [28]. Vitamin D has complex effects on pulmonary cell biology and immunity with impact on inflammation, host defense, wound healing, repair, and other processes modulating the function of various immune cells. Interestingly, vitamin D may protect from developing respiratory infections that could serve as trigger for a deterioration of asthma [29] and is potentially capable of overcoming the poor glucocorticoid responsiveness in severe asthmatics by upregulation of IL-10 production from CD4+ T cells [30]. Accordingly, showing 25(OH) Vit D lower levels in children with AR and AAR, we underline that a lack of 25(OH) Vit D levels is able to increase the risk of allergic disease of the airways in children. However, the underlying mechanisms by which vitamin D modulates the pathogenesis of asthma are not clear and further studies might be necessary to clarify its involvement in the immunology of airway disease.

CD4+ T cells producing Th2-type cytokines are thought to have a pivotal role in orchestrating the recruitment and activation of these effector cells of the allergic response [31]. IL-31 is a cytokine produced by CD4+ T cells playing an important role in allergic inflammation often showing a potent immunological link with IL-33 [32]. IL-31 receptor IL-31RA, oncostatin M receptor, and IL-33 receptor ST2/IL1RL1 are also related with the immunopathological mechanisms of allergic diseases [33–35]. IL-33 exerts its function through the ST2 receptor, a critical component of Th2 response [36], generating the production of IL-31 [33]. IL-31 signals act through a receptor composed of IL-31 receptor A and oncostatin M receptor promoting the cell activation during allergic diseases [35]. Accordingly, we observed statistically significant higher levels of IL-31 and IL-33 in children with AAR than in HC and, though without a statistical difference, in AR than in HC. In both AR and AAR the levels of IL-31 and IL-33 positively correlated. These findings underline that IL-31 and IL-33 are linked and might be involved in the pathogenesis of allergic rhinitis and asthma with a potential role in the progression of allergic disease in asthma. In this scenario, we are able to hypothesize that IL-31 and the related IL-33 activity might be linked with vitamin D in allergic disease. Nevertheless, we observed that the levels of IL-31 and IL-33 were not statistically different in patients with AAR and AR with deficient, insufficient, and sufficient levels of 25(OH) Vit D. Indeed the fact that we even observed higher levels of IL-31 and IL-33 in patients with AAR showing insufficient and sufficient 25(OH) Vit D levels further underlined the lack of relationship between 25(OH) Vit D IL-31 and IL-33 in allergic disease. Vitamin D is able to modulate the function of CD4+ T cells particularly reversing the defective induction of IL-10-secreting regulatory T cells in glucocorticoid-resistant asthma patients [30]. Nevertheless, our results suggest that it is not involved in IL-33/IL-31 Th2-type cytokines activity implicated in bronchial and nasal allergic disease. Finally, in accordance with our results, it was observed that serum IL-31 is related to oncostatin M and vitamin D3 levels in normal donors, while no correlation with vitamin D was observed in patients with AD [35]. In this scenario we can speculate that our results might underline that IL-31 and the related IL-33 are not Th2 cytokines in the classical sense as previously described [32]. Particularly, we suggest that since IL-31 is likely to be expressed by a number of cells in the allergic situations in which IL-4 is present and promotes Th2-driven inflammation [36], it contributes in allergic reaction involving the vitamin D deficiency only partially. However further studies might be necessary to clarify the mechanism how vitamin D is able to control the activity of CD4+ T cells and the related Th2-type cytokines in the pathogenesis of allergic disease.

Vitamin D deficiency has been blamed as one cause of increased asthma prevalence in the last decades [37]. Epidemiologic studies have also shown that maternal vitamin D intake during pregnancy protects from wheezing in childhood [38]. A recent clinical investigation showed that high vitamin D levels are associated with better lung function, less airway hyperresponsiveness, and improved glucocorticoid response [39]. Vitamin D deficiency/insufficiency is common in children with mild to moderate persistent asthma and is associated with higher odds of severe exacerbation [40]. Accordingly, we showed that children with concomitant allergic rhinitis and asthma, having deficient or insufficient 25(OH) Vit D, had marked characteristic of disease in terms of exacerbation number in the last year and in terms of disease duration (yrs). Our findings support the concept that the intake of lower levels of vitamin D in asthmatic children is able to influence a worse course of the allergic disease, representing a minor favorable outcome for children with allergic rhinitis to develop concomitant asthma.

There is little knowledge about clinical variables associated with vitamin D deficiency/insufficiency in asthmatic children, with numerous controversies in the literature regarding the role of vitamin D in allergy. One hypothesis for the increasing prevalence of asthma involves vitamin D. Some have argued that vitamin D has more of a deleterious effect on allergic pathogenesis [3]. Although multiple studies have examined maternal vitamin D status and subsequent wheezing in offspring [41], there are limited data on vitamin D levels in children with asthma, as well as on which features of asthma are associated with vitamin D levels. However, a recent large epidemiological study looking at data from a British cohort showed an association between elevated IgE levels and very low (<25 nmol/L) or very high 25-hydroxyvitamin D3 (>135 nmol/L) levels. Nevertheless, a significant but nonlinear relationship was found between 25-hydroxyvitamin D3 status and IgE levels. It therefore appears that too little or too much vitamin D may predispose to the development of allergic immune responses [42]. In this scenario, despite the controversy in the literature and despite our small sample of children with concomitant allergic asthma and rhinitis, we showed that the levels of 25(OH) Vit D inversely correlated with total allergy sIgE score and with total atopy index, further demonstrating a negative effect of lower levels of vitamin D on respiratory allergic diseases. This finding clearly suggests that low levels of 25(OH) Vit D are associated to the degree of multiple allergen sensitizations in the patients. Finally, we showed that children with AAR with positive sIgE to dust mites had lower levels of 25(OH) Vit D. These findings further underline and support the concept that vitamin D might be necessary as an adjunct to subcutaneous allergen immunotherapy in asthmatic children sensitized to house dust mite as previously described [43].

In conclusion, this study demonstrated the effect of vitamin D levels on the related outcomes of asthma and of allergic phenotypes of the disease, with a particular link with the host dust mite sensitization. Since most allergies start in childhood, vitamin D deficiency or insufficiency in childhood might influence initiation of allergy targeting unclear and little known immunologic aspect of the disease. Further clinical studies might clarify the link between vitamin D and asthma and allergic immunologic features.

## Figures and Tables

**Table 1:** Demographic, clinical, and atopic characteristic of the subjects.

	Control (HC)	Allergic rhinitis (AR)	Allergic asthma rhinitis (AAR)	Overall *P* value
Age, (years) *range *	10.6 *(±2.2)* *8–12 *	10.3 *(±2.1)* *8–11 *	10.7 *(±2.5)* * 9–13 *	*ns *
Sex, *n* *(male/female) *	28 (17/11)	11 (8/3)	35 (22/13)	*ns *
Body mass index, (kg/m^2^) *range *	25.1 *(±10.3)* *17.8–32.3 *	18.6 *(±4.8)* *14.8–19.7 *	19.6 *(±3.0)* *17.1–21.3 *	*ns *
FEV1 (% predicted)	110.0 *(±9.0) *	107.7 *(±16.7) *	93.5 *(±12.6) *	*<0.0001 *
FVC (% predicted)	95.5 *(±19) *	106 *(±118) *	92.9 *(±12.1) *	*<0.0001 *
PEF (% predicted)	80.6 *(±14.6) *	79.7 *(±18.6) *	60.1 *(±15.7) *	*<0.0001 *
SPT, any positive, *n* (%)	0	100	100	*<0.0001 *
Total IgE (IU/mL), *(geometric mean)* log* tot IgE *	39.7 *1.60 (±0.21) *	293.2 *2.47 (±0.47) *	320.5 *2.51 (±0.41) *	*<0.0001* *<0.0001 *
Total allergen sIgE score	0	6.0 *(±3.3) *	6.7 *(±4.7) *	*<0.0001 *
Rast to dust mites	0	2.4 *(±2.0) *	2.1 *(±2.0) *	*<0.0001 *
Exacerbations (last year)	0	NA	2.1 *(±1.5) *	*<0.0001 *
Disease duration (yrs)	0	4.2 *(±2.0) *	5.6 *(±2.5) *	*<0.0001 *

Data are presented as mean ± standard deviation unless otherwise stated. FEV1 = forced expiratory volume in 1 s; FVC = forced vital capacity; PEF = peak expiratory flow; SPT = skin prick test. Statistical analysis for multiple comparisons was calculated by Kruskal-Wallis test.

**Table 2:** Frequency distribution of vitamin D status classes among HC, AR, and AAR groups.

	HC *n* = 28	AR *n* = 11	AAR *n* = 35	*P* value (*χ* ^2^)
Vitamin D status				<0.009
Deficient (<20 ng/mL), *n* (%)	1 (3.6%)	2 (18.2%)	6 (17.1%)	
Insufficient (20–30 ng/mL), *n* (%)	10 (35.7%)	7 (63.6%)	22 (62.9%)	
Sufficient (>30 ng/mL), *n* (%)	17 (60.7%)	2 (18.2%)	7 (20.0%)	
